# Full blood count dynamics in immunologically naïve individuals with mild COVID-19: A prospective community cohort study

**DOI:** 10.1371/journal.pone.0353142

**Published:** 2026-07-09

**Authors:** Seran Hakki, Sean Nevin, Emily Conibear, Kieran J. Madon, Joe Fenn, Jakob Jonnerby, Nieves Derqui, Aleksandra Koycheva, Rhia Kundu, Hamish Houston, Timesh D. Pillay, Alexandra L. Kondratiuk, Janakan Sam Narean, Kimone L. Fisher, Robert Varro, Constanta Luca, Samuel Evetts, Peter Kelleher, Onn Min Kon, Graham P. Taylor, Ajit Lalvani

**Affiliations:** 1 NIHR Health Protection Research Unit in Respiratory Infections, National Heart and Lung Institute, Imperial College London, London, United Kingdom; 2 Department of Infectious Diseases, Imperial College London, London, United Kingdom; 3 National Heart and Lung Institute, Imperial College London, London, United Kingdom; 4 Section of Virology, Department of Infectious Disease, Imperial College London, London, United Kingdom; Instituto Nacional de Salud Pública: Instituto Nacional de Salud Publica, MEXICO

## Abstract

**Purpose:**

Full blood count (FBC) provides a range of cellular haematological parameters and serves as a routinely available basic immune profile. While FBC has been widely used to monitor moderate-to-severe COVID-19 in hospitalised settings, its temporal dynamics in mild, community-managed cases remain poorly characterised, despite these constituting the majority of global infections.

**Methods:**

In a prospective cohort study, we tracked the cellular haematological profiles of 93 recently exposed, immunologically naïve individuals with mild COVID-19 and no underlying co-morbidities, recruited to the Integrated Network for Surveillance, Trials and Investigations into COVID-19 Transmission (INSTINCT) study. Blood samples were collected on D0, D7, D14 and D28 and subsequently aligned to infection-timepoints based on the day of first detected PCR positivity, and analysed using mixed-effects models.

**Results:**

Over 30% of cases exhibited transient, clinically defined neutropenia (mean (95% CI): First PCR-Positive (FP) 2.47x10^9^/L (2.26–2.68) vs convalescence 3.34 x10^9^/L (2.98–3.7); p = 0.0013). Over 20% exhibited lymphopenia (FP 1.38x10^9^/L (1.28–1.48) vs convalescence 1.79x10^9^/L (1.79–2.01); p = 0.0013). Additionally, we observed a notable elevation in platelet count, peaking approximately two weeks after initial infection (mean (95% CI): FP + 14 283x10^9^/L (254–311) vs convalescence 237x10^9^/L (222–252); p = 0.0013).

**Conclusions:**

Transient neutropenia and lymphopenia occurred in approximately one-third and one-fifth of mild COVID-19 cases, respectively, and were followed by a delayed increase in platelet count. This study provides a descriptive, prospective dataset of full blood count parameters spanning early infection to convalescence in immunologically naïve individuals with mild SARS-CoV-2 infection. These data may support mathematical modelling of within-host cellular haematological dynamics and have potential clinical relevance for understanding typical trajectories of routine blood parameters during mild disease.

## Introduction

Clinical outcomes of SARS-CoV-2 infection vary widely and are largely determined by the host immune response. Full blood count (FBC) provides a window into a range of haematological parameters including a routinely available basic immune profile. Many cross-sectional studies [1,2], as well as some that have sampled longitudinally [3,4], have used FBCs in hospitalised patients with moderate-to-severe COVID-19 to inform prognostic tools. There is, however, a dearth of longitudinal studies in mild community cases, which account for most transmission globally. Moreover, understanding the dynamic host responses from the earliest timepoints after exposure when a wholly new virus enters an immunologically naïve global population is of inherent biological interest and is clinically relevant for recognising the typical course of infection, enabling timely interventions. We therefore present a detailed serially sampled dataset of cellular haematological profiles based on full blood counts, from recently infected, immunologically naïve, mild COVID-19 cases, highlighting transient neutropenia, lymphopenia, and delayed platelet changes, which may have potential clinical relevance and provide a resource for future modelling studies.

## Materials & methods

### Study design

INSTINCT (*Integrated Network for Surveillance, Trials and Investigations into COVID-19 Transmission*), a longitudinal prospective community study (20/NW/0231) [5], recruited London households with a PCR-positive COVID-19 case identified by the UK Health Security Agency contact tracing program between 27/05/2020–23/03/2021, encompassing the Pre-Alpha and Alpha waves. Ethics approval was obtained from the Health Research Authority (Research Ethics Committee reference 20/NW/0231). Written informed consent was obtained from all participants, including minors with consent from parents or guardians, as documented.

Nurses visited households on the day of enrolment (day [D]0), D7, D14 and D28 and took blood and upper respiratory tract (URT) swabs from participants. Participants self-sampled an additional URT swab on D4. Swabs were processed for RT-PCR, and blood samples were processed for quantitation of SARS-CoV-2-specific antibodies as previously described [5]. FBC analysis was performed on blood samples using the Alinity h-series Integrated Haematology System (Abbott Laboratories, Illinois, United States).

**Fig 1:**
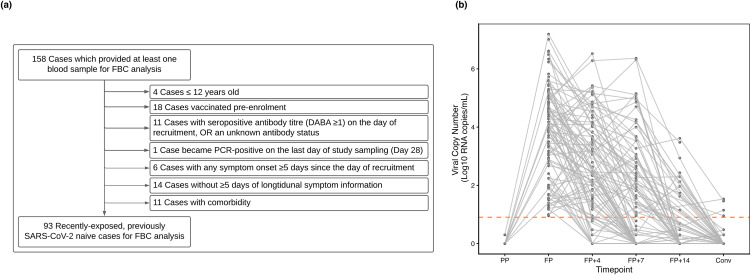
(a) Stepwise exclusion criteria used in the cohort selection of 93 recently-exposed, SARS-CoV-2 naïve COVID-19 cases and (b) SARS-CoV-2 RNA viral trajectories during acute infection. Cases were excluded for age (<12 years), prior vaccination, pre-existing antibody seropositivity, late PCR conversion or symptom onset after recruitment, inadequate symptom reporting, or presence of confounding comorbidities. (b) The orange dotted line represents the PCR-positivity threshold of eight viral RNA copies/ml.

To account for heterogeneity in the day of enrolment relative to the day of acquiring detectable infection, sampling timepoints were realigned relative to the day of the first PCR-positive study sample (Fig 1b). “Pre-positive” (PP) refers to blood samples taken prior to the case testing PCR-positive, whilst “First PCR-positive” (FP) refers to the blood samples taken when the case first tested PCR-positive in the study. “FP+7” and “FP+14” refers to samples taken seven- and fourteen-days post-FP respectively. Samples collected approximately four weeks after enrolment are denoted “Convalescent”.

We applied a number of exclusion criteria to ensure the quality and consistency of FBC data used in our analyses. From an initial cohort of 158 participants, we excluded individuals ≤12 years old, those with prior SARS-CoV-2 vaccination or seropositivity indicative of prior infection, individuals who did not become PCR positive within the first two study timepoints, those with symptom onset ≥5 days from the day of recruitment, those with ≤5 days of consecutively collected longitudinal symptom data and individuals with comorbidities likely to affect blood count parameters. This resulted in a final study cohort of 93 participants.

### Statistical analysis

Statistical analysis was performed in R (version 4.1.1) and GraphPad Prism. In GraphPad Prism, the mixed-effects model was specified with *timepoint* as a fixed effect, and *subject* as a random effect (random intercepts with additional random variation at each timepoint nested within subjects). Parameters were estimated using restricted maximum likelihood (REML), which accommodates missing values without requiring imputation. Covariance among repeated measures was modelled implicitly through the random effects, and Prism does not allow the specification of alternative covariance structures.

Histogram and ridge density plots were inspected and visually confirmed for approximate normality of the full blood count parameters across timepoints (S1 Fig, S2 Fig in S1 File) with the exception of eosinophils and basophils, which exhibited skewed distributions. This is likely due to low counts frequently falling below the lower limit of detection, with such values assigned to the LLOD.

To provide a measure of estimate reliability, a precision analysis was performed for key full blood count parameters. For each timepoint, the half-width of the 95% confidence interval was calculated as $HalfwidthCI=t_{\text{0}.\text{9}\text{7}\text{5},n-\text{1}}*\left(s/sqrt(n)\right)$, where *s* is the observed standard deviation and *n* the sample size, reported in S2 Table in S1 File. This approach highlights that timepoints with smaller groups, such as the pre-positive and FP + 14 cohorts, yield wider confidence intervals and therefore less precise estimates.

## Results & discussion

A total of 93 recently exposed, SARS-CoV-2–naïve, PCR-positive individuals with mild ambulatory disease and no known underlying health conditions were included for full blood count analysis following application of the study exclusion criteria (Fig 1a). The median day of symptom onset relative to enrolment was three days (range –5–0), and two participants were asymptomatic. Demographic characteristics of the study cohort are summarized in S1 Table in S1 File.

**Fig 2:**
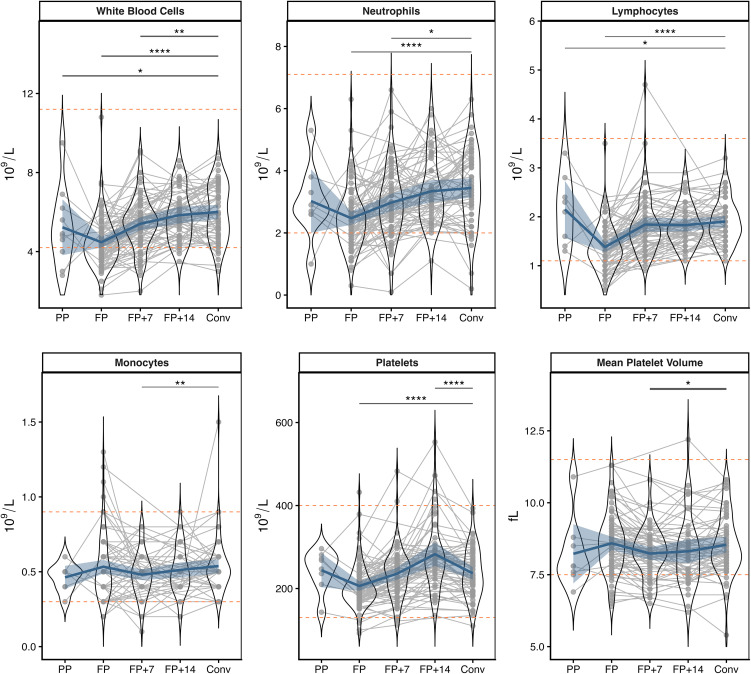
Longitudinal full blood count (FBC) dynamics following recent SARS-CoV-2 infection in 93 recently-exposed, SARS-CoV-2 naïve COVID-19 cases. Longitudinal violin plot of FBC dynamics. Mean and 95% confidence limits are depicted by the blue line and shaded areas respectively, with individual case trajectories in grey. Orange dotted lines indicate the thresholds of clinically-defined normal ranges from each FBC parameter. Maximum count: PP, pre-positive timepoint (n = 10); FP, first study-positive (n = 90); FP + 7, seven days after first study-positive (n = 70); FP + 14 fourteen days after first study-positive (n = 41); Conv, convalescent (n = 64). P values were calculated using a mixed-effects model (REML) and adjusted for multiple comparisons using Dunnett’s method, comparing each timepoint against the convalescent timepoint. To account for multiple FBC parameters, P-values were corrected using the Bonferroni method (S3 Table in S1 File). Summary statistics including means and half-width and 95% confidence limits are listed in S2 Table in S1 File. The remainder of the FBC parameters can be seen in S3 Fig in S1 File (*p < 0.05, **p < 0.01, ***p < 0.001, ****p < 0.0001).

FBC parameters at all timepoints were compared to the convalescent timepoint to investigate changes during infection and recovery. We observed transient leukopenia driven principally by neutropenia (count < 2x10^9^/L) and lymphopenia (count < 1.1x10^9^/L) at the time of first PCR-positivity (FP) (Fig 2, S2 Table in S1 File). This was reflected at the individual level, with lower neutrophil counts at FP than convalescence in 51/60 participants and lower lymphocyte counts in 55/60 participants, consistent with overall recovery of these cell populations during convalescence. Remarkably, almost a third of the cases, all of which had no known underlying health conditions, developed clinically-relevant neutropenia (28/89, 31.5%) (S4 Table in S1 File), with 20% (5/25) of these cases maintaining clinically-relevant neutropenia one week later. These observations in otherwise healthy adults provide a reference for early haematological responses in mild COVID-19 and may have potential clinical relevance for individuals with pre-existing immunosuppression [6].

The transient decline in lymphocyte and neutrophil cell counts likely resulted from cell migration from the periphery to the infection site. Indeed, in severe COVID-19 cases, single-cell RNA sequencing on nasopharyngeal and bronchial samples demonstrates mucosal enrichment of neutrophils compared to healthy controls, indicating tissue redistribution [7]. Whilst transient lymphopenia is recognised in mild COVID-19 [4], the occurrence of early neutropenia has remained largely unreported, except as a chance finding in patients receiving clozapine therapy for mental illness, who developed COVID-19 during the study period [8]. In that cohort, most cases were mild (24/31, 77.4%) also, with a minority experiencing moderate disease (7/31, 22.6%), supporting the relevance of our observations within the non-severe clinical spectrum. This observation contrasts the hallmark of elevated neutrophil counts observed in hospitalised patients, which typically manifest later following exposure compared to our cohort. The rapid restoration of neutrophil counts to baseline levels therefore serves as a pivotal marker of successful recovery. Conversely, dysregulation resulting in an overproduction of neutrophils underscores an underlying mechanism which leads to severe disease [9].

Scavone *et al.* observed mild abnormalities in platelet parameters, including increased circulating activated platelets among hospitalised COVID-19 patients with no evidence of pneumonia [10]. However, the temporal dynamics of platelets during early ambulatory SARS-CoV-2 infection have been little characterised. We report a modest decline in platelet count during the initial phase of mild infection (mean (95% CI): FP 206x10^9^/L (195–217) vs convalescence 237x10^9^/L (222–252); p = 0.0013), followed by an elevation above convalescent levels approximately two weeks post-infection (mean (95% CI): FP + 14 283x10^9^/L (254–311) vs convalescence 237x10^9^/L (222–252); p = 0.0013) (Fig 2, S2 Table in S1 File), with absolute thrombocytosis observed in 4/40 cases at FP + 14 (S4 Table in S1 File).

We suggest that the early transient thrombocytopenia (count < 130x10^9^/L) may reflect activated platelet migration to mucosal sites. Given that activated platelets reduce in size as they release their granules, this hypothesis is supported by the significant decrease in mean platelet volume (MPV) at FP + 7 (Fig 2, S2 Table in S1 File). Cases with the greatest reduction in MPV at FP + 7 also showed the largest platelet count increase a week later (S4 Fig, in S1 File Pearson’s correlation, p = 0.0003), indicating a possible relationship between platelet activation and reactive thrombocytosis. Interestingly, platelet count has also been shown to peak around the eighth day of hospitalisation in severe COVID-19 patients [11], which temporally aligns with FP + 14 in our study of mild disease. These observations highlight dynamic changes in platelet counts and MPV during mild COVID-19.

In severe COVID-19 cases, lung and endothelial damage leads to excessive platelet aggregation and microthrombi formation, worsening the condition [12]. Whilst venous-thromboembolism (VTE) is associated with hospitalised COVID-19 patients, it has also recently emerged as a significant complication following mild COVID-19 infection, with a 2.74-fold higher incidence in mild cases compared to matched healthy controls, and the highest risk occurring within 30 days of COVID-19 diagnosis [13]. In this context, the delayed elevation in platelet counts observed in our cohort, although generally within the normal clinical range, may have potential clinical relevance for understanding thrombotic risk. Previous studies have considered anti-platelet therapeutics, such as aspirin, in COVID-19, highlighting the clinical interest in platelet dynamics [14].

Our study has several limitations. Our cohort primarily consisted of white individuals; hence the findings may not be generalizable to more diverse populations. While FBC results offer a broad overview of the immune profile, more in-depth techniques are required to further delineate leukocyte subsets. For example, disease severity in COVID-19 has been previously associated with ratios of distinct monocyte subsets and their activation patterns, suggesting probable differences in the temporal kinetics amongst these subsets [15]. As we were limited to only measuring total monocyte count, this likely accounts for the absence of significant differences in the temporal kinetics of monocyte count at the early timepoints. We acknowledge that sample sizes varied across timepoints, a limitation reflecting both the realities of clinical research and our study design. The Day-14 sampling timepoint was an optional additional visit, resulting in fewer participants attending at that timepoint, and the FP + 14 timepoint similarly had a smaller sample size. Notwithstanding the smaller sample size at FP + 14, the platelet increase observed at FP + 14 was highly significant (p < 0.0001). As the reduced Day-14 visit sample size was expected due to the Day-14 visit being optional, and not due to clinical factors, missing data is assumed to be missing at random (MAR). Finally, all cases were recruited in London during the pre-Alpha and Alpha waves. This constrains the generalisability of results to later variants and circulating strains, and the findings cannot be directly extended to Omicron or future SARS-CoV-2 waves. Despite these limitations, the dataset provides a well-characterised longitudinal record of haematological changes in immunologically naïve individuals with mild COVID-19 and may serve as a valuable resource for future modelling of within-host dynamics, comparative studies, hypothesis generation, and understanding early immune responses in the context of emerging pathogens.

## Conclusion

Entry of a new virus into immunologically-naïve hosts is a rare natural experiment and one which cannot be repeated for SARS-CoV-2 due to widespread antigen exposure. Our study has revealed hitherto unappreciated dynamic perturbations in FBC parameters, in particular transient neutropenia followed by a change in platelet volume and elevation in platelet count. These data constitute a resource for future modelling and comparative studies of haematological dynamics and may have potential clinical relevance for understanding typical trajectories of routine blood parameters in mild COVID-19.

## Supporting information

S1 File This file contains all supplementary materials associated with this study, including Tables S1–S4 and Figures S1–S4. (DOCX)

## References

[1] Zhu B, Feng X, Jiang C, Mi S, Yang L, Zhao Z, et al. Correlation between white blood cell count at admission and mortality in COVID-19 patients: A retrospective study. BMC Infect Dis. 2021;21(1):574. doi: 10.1186/s12879-021-06277-3 34126954 PMC8202964

[2] Antunez Muiños PJ, López Otero D, Amat-Santos IJ, López País J, Aparisi A, Cacho Antonio CE, et al. The COVID-19 lab score: An accurate dynamic tool to predict in-hospital outcomes in COVID-19 patients. Sci Rep. 2021;11(1):9361. doi: 10.1038/s41598-021-88679-6 33931677 PMC8087839

[3] Burke H, Freeman A, O’Regan P, Wysocki O, Freitas A, Dushianthan A, et al. Biomarker identification using dynamic time warping analysis: A longitudinal cohort study of patients with COVID-19 in a UK tertiary hospital. BMJ Open. 2022;12(2):e050331. doi: 10.1136/bmjopen-2021-050331 35168965 PMC8852240

[4] Ouyang SM, Zhu HQ, Xie YN, Zou ZS, Zuo HM, Rao YW. Temporal changes in laboratory markers of survivors and non-survivors of adult inpatients with COVID-19. BMC Infect Dis. 2020;20:952. doi: 10.1186/s12879-020-05678-0 33308159 PMC7729703

[5] Houston H, Hakki S, Pillay TD, Madon K, Derqui-Fernandez N, Koycheva A, et al. Broadening symptom criteria improves early case identification in SARS-CoV-2 contacts. Eur Respir J. 2022;60(1):2102308. doi: 10.1183/13993003.02308-2021 34824057 PMC8620106

[6] Adachi E, Saito M, Nagai H, Ikeuchi K, Koga M, Tsutsumi T, et al. Transient depletion of T cells during COVID-19 and seasonal influenza in people living with HIV. J Med Virol. 2022;94(5):1789–91. doi: 10.1002/jmv.27543 34978090 PMC9015586

[7] Chua RL, Lukassen S, Trump S, Hennig BP, Wendisch D, Pott F, et al. COVID-19 severity correlates with airway epithelium-immune cell interactions identified by single-cell analysis. Nat Biotechnol. 2020;38(8):970–9. doi: 10.1038/s41587-020-0602-4 32591762

[8] Vallecillo G, Marti-Bonany J, Robles MJ, Fortuny JR, Lana F, Pérez V. Transient drop in the neutrophil count during COVID-19 regardless of clozapine treatment in patients with mental illness. Rev Psiquiatr Salud Ment (Engl Ed). 2022;15(2):134–7. doi: 10.1016/j.rpsmen.2022.06.006 35840279 PMC9274211

[9] Meizlish ML, Pine AB, Bishai JD, Goshua G, Nadelmann ER, Simonov M, et al. A neutrophil activation signature predicts critical illness and mortality in COVID-19. Blood Adv. 2021;5(5):1164–77. doi: 10.1182/bloodadvances.2020003568 33635335 PMC7908851

[10] Scavone M, Ghali C, Calogiuri M, Sala M, Bossi E, Mencarini T, et al. Impairment of platelet function in both mild and severe COVID-19 patients. Br J Haematol. 2023;203(4):656–67. doi: 10.1111/bjh.19062 37615207

[11] Barrett TJ, Bilaloglu S, Cornwell M, Burgess HM, Virginio VW, Drenkova K, et al. Platelets contribute to disease severity in COVID-19. J Thromb Haemost. 2021;19(12):3139–53. doi: 10.1111/jth.15534 34538015 PMC8646651

[12] Palladino M. Complete blood count alterations in COVID-19 patients: A narrative review. Biochem Med (Zagreb). 2021;31(3):030501. doi: 10.11613/BM.2021.030501 34658642 PMC8495616

[13] Raisi-Estabragh Z, Cooper J, Salih A, Raman B, Lee AM, Neubauer S, et al. Cardiovascular disease and mortality sequelae of COVID-19 in the UK Biobank. Heart. 2022;109(2):119–26. doi: 10.1136/heartjnl-2022-321492 36280346 PMC9811071

[14] Zareef R, Diab M, Al Saleh T, Makarem A, Younis NK, Bitar F, et al. Aspirin in COVID-19: Pros and Cons. Front Pharmacol. 2022;13: 849628. doi: 10.3389/fphar.2022.849628 35370686 PMC8965577

[15] Schulte-Schrepping J, Reusch N, Paclik D, Baßler K, Schlickeiser S, Zhang B, et al. Severe COVID-19 Is marked by a dysregulated myeloid cell compartment. Cell. 2020;182(6):1419-1440.e23. doi: 10.1016/j.cell.2020.08.001 32810438 PMC7405822

