# Supplementary material for: Full blood count dynamics in immunologically naïve individuals with mild COVID-19: A prospective community cohort study
Source: PLoS One. 2026 Jul 9;21(7):e0353142. doi: 10.1371/journal.pone.0353142 (PMC13349189; doi:10.1371/journal.pone.0353142)
Supplement: S1 File — (DOCX) [file pone.0353142.s001.docx]

**Supporting Information**

**Full blood count dynamics in immunologically naïve individuals with mild COVID-19: a prospective community cohort study**

Seran Hakki*^1^, Sean Nevin*^1^, Emily Conibear**^1^, Kieran J Madon**^1^, Joe Fenn^1^, Nieves Derqui^1^, Aleksandra Koycheva^1^, Jakob Jonnerby^1^, Rhia Kundu^1^, Hamish Houston^1^, Timesh D Pillay^1^, Alexandra L Kondratiuk^1^, Janakan Sam Narean^1^, Kimone L Fisher^1^, Robert Varro^1^, Constanta Luca^1^, Samuel Evetts^1^, Prof. Peter Kelleher^2^, Prof. Onn Min Kon^3^, Prof. Graham P Taylor^4^, Prof. Ajit Lalvani^1^.

* S.H and S.N contributed equally to this study (joint-first equal contributions),

** E.C and K.J.M contributed equally to this study (joint-second equal contributions).

**Affiliations**

**1** NIHR Health Protection Research Unit in Respiratory Infections, National Heart and Lung Institute, Imperial College London, London, UK.

**2** Department of Infectious Diseases, Imperial College London, London, UK.

**3** National Heart and Lung Institute, Imperial College London, London, UK

**4** Section of Virology, Department of Infectious Disease, Imperial College London, London, UK.

**Corresponding author:**

Dr Seran Hakki

Dr Victor Phillip Dahdaleh Building (VPD),

Imperial College Hammersmith Campus,

Du Cane Road, London,

W12 0NN

s.hakki@imperial.ac.uk

**Supplementary Tables**

**Table S1:** The demographic characteristics of the 93 recently-exposed, SARS-CoV-2 naïve cases for FBC analysis.

**Table S2:** Summary statistics of the temporal dynamics of full blood count parameters following recent SARS-CoV-2 infection.

**Table S3:** Fig2 Mixed-effects model statistical test results.

**Table S4:** Proportion of study participants that presented with full blood count parameters outside of the normal clinical range.

**Supplementary Figures**

**Fig.S1: Histograms of full blood count (FBC) variables across all timepoints.**

**Fig.S2: Ridge plots of full blood count (FBC) variables by infection timepoint.**

**Fig.S3:** The remainder of the longitudinal full blood count parameter dynamics in 93 cases.

**Fig.S4:** Correlation between mean platelet volume (MPV) at FP+7 and Platelet count at FP+14.

**Supplementary Tables**

**Table S1:** **The demographic characteristics of the 93 recently-exposed, SARS-CoV-2 naïve cases for FBC analysis.** *BMI was not recorded for cases under the age of eighteen. †Non-White ethnic groups included Indian sub-continent Indian (n=4), Indian subcontinent Pakistani (n=2), Indian sub-continent Afghanistan (n=1), Mixed White & Asian (n=3), Black Caribbean (n=3), Asian Chinese (n=1), Mixed White & Asian (n=3), Mixed White & Middle Eastern (n=1), and Hispanic (n=1). ‡ Three cases of non-systemically managed asthma and two cases of clinically diagnosed obesity.

| **Sex** | Female (%) | 43 (46.2) |
| --- | --- | --- |
|  | Male (%) | 50 (53.8) |
| **Age in years** | Median (IQR) | 36 (26-46) |
|  | 13 - 20 (%) | 12 (12.9) |
|  | 21 - 30 (%) | 23 (24.7) |
|  | 31 - 40 (%) | 23 (24.7) |
|  | 41 - 50 (%) | 21 (22.6) |
|  | 51 - 60 (%) | 8 (8.6) |
|  | ≥ 61 (%) | 4 (4.3) |
|  | Unknown (%) | 2 (2.2) |
| **Body mass index*** | Median (IQR) | 23.9 (20.9-26.8) |
|  | Underweight (%) | 4 (3.8) |
|  | Normal (%) | 42 (39.6) |
|  | Overweight (%) | 24 (22.6) |
|  | Obese (%) | 5 (4.7) |
|  | Morbidly obese (%) | 3 (2.8) |
|  | Unknown (%) | 15 (14.2) |
| **Ethnicity** † | White (%) | 79 (84.9) |
|  | Non-white (%) † | 14 (15.1) |
| **Co-morbidities** ‡ | Yes (%) ‡ | 5 (5.4) |
|  | No (%) | 87 (93.5) |
|  | Unknown | 1 (1.1) |

**Table S2: Summary statistics of the temporal dynamics of full blood count parameters following recent SARS-CoV-2 infection.** Count, mean, confidence intervals (CI) of the mean, half width CI, median and interquartile ranges of each cell type at each timepoint are listed. Maximum count: PP, pre-positive timepoint (n=10); FP, first study-positive (n=90); FP+7, seven to ten days after first study-positive (n=70); FP+14 fourteen days after first study-positive (n=41); Conv, convalescent (n=64).

| **WHITE BLOOD CELLS (10^9^/L)** | | **Pre-Positive**  **(PP)** | **First study-positive**  **(FP)** | | **First study-positive  plus 7 days**  **(FP+7)** | | **First study-positive  plus 14 days**  **(FP+14)** | | **Convalescence**  **(Conv)** | |  |  |
| --- | --- | --- | --- | --- | --- | --- | --- | --- | --- | --- | --- | --- |
| Count | | 10 | 90 | | 70 | | 41 | | 64 | |  |  |
| Mean | | 5.23 | 4.47 | | 5.41 | | 5.85 | | 6.01 | |  |  |
| 95% CI | | 3.82-6.64 | 4.21-4.74 | | 5.07-5.74 | | 5.42-6.27 | | 5.65-6.36 | |  |  |
| Half width CI | | 1.41 | 0.27 | | 0.33 | | 0.42 | | 0.36 | |  |  |
| Median | | 4.85 | 4.4 | | 5.45 | | 5.7 | | 5.9 | |  |  |
| Interquartile Range | | 3.75-6.38 | 3.7-5.2 | | 4.58-6.08 | | 4.75-7 | | 4.83-7.38 | |  |  |
| **NEUTROPHILS (10^9^/L)** | | **PP** | **FP** | | **FP+7** | | **FP+14** | | **Conv** | |  |  |
| Count | | 8 | 89 | | 70 | | 41 | | 63 | |  |  |
| Mean | | 3.03 | 2.47 | | 2.98 | | 3.34 | | 3.45 | |  |  |
| 95% CI | | 2-4.05 | 2.26-2.68 | | 2.7-3.26 | | 2.98-3.7 | | 3.14-3.75 | |  |  |
| Half width CI | | 1.02 | 0.21 | | 0.28 | | 0.36 | | 0.31 | |  |  |
| Median | | 2.8 | 2.4 | | 2.9 | | 3.1 | | 3.4 | |  |  |
| Interquartile Range | | 2.6-3.68 | 1.8-2.9 | | 2.2-3.65 | | 2.35-4.2 | | 2.6-4.4 | |  |  |
| **LYMPHOCYTES (10^9^/L)** | | **PP** | **FP** | | **FP+7** | | **FP+14** | | **Conv** | |  |  |
| Count | | 8 | 89 | | 70 | | 41 | | 63 | |  |  |
| Mean | | 2.15 | 1.38 | | 1.84 | | 1.83 | | 1.9 | |  |  |
| 95% CI | | 1.57-2.73 | 1.28-1.48 | | 1.7-1.99 | | 1.7-1.96 | | 1.79-2.01 | |  |  |
| Half width CI | | 0.58 | 0.1 | | 0.15 | | 0.13 | | 0.11 | |  |  |
| Median | | 2.2 | 1.3 | | 1.75 | | 1.8 | | 1.8 | |  |  |
| Interquartile Range | | 1.45-2.7 | 1.1-1.6 | | 1.5-2.13 | | 1.55-2.1 | | 1.6-2.2 | |  |  |
| **MONOCYTES (10^9^/L)** | | **PP** | **FP** | | **FP+7** | | **FP+14** | | **Conv** | |  |  |
| Count | | 8 | 89 | | 70 | | 41 | | 63 | |  |  |
| Mean | | 0.46 | 0.53 | | 0.48 | | 0.51 | | 0.54 | |  |  |
| 95% CI | | 0.39-0.54 | 0.49-0.58 | | 0.44-0.52 | | 0.46-0.56 | | 0.49-0.58 | |  |  |
| Half width CI | | 0.08 | 0.05 | | 0.04 | | 0.05 | | 0.05 | |  |  |
| Median | | 0.5 | 0.5 | | 0.5 | | 0.5 | | 0.5 | |  |  |
| Interquartile Range | | 0.4-0.5 | 0.4-0.7 | | 0.4-0.6 | | 0.4-0.6 | | 0.4-0.6 | |  |  |
| **EOSINOPHILS (10^9^/L)** | | **PP** | **FP** | | **FP+7** | | **FP+14** | | **Conv** | |  |  |
| Count | | 5 | 84 | | 67 | | 41 | | 60 | |  |  |
| Mean | | 0.06 | 0.09 | | 0.11 | | 0.13 | | 0.16 | |  |  |
| 95% CI | | -0.05-0.17 | 0.07-0.11 | | 0.08-0.13 | | 0.11-0.16 | | 0.13-0.2 | |  |  |
| Half width CI | | 0.11 | 0.02 | | 0.02 | | 0.03 | | 0.03 | |  |  |
| Median | | 0 | 0.1 | | 0.1 | | 0.1 | | 0.1 | |  |  |
| Interquartile Range | | 0-0.15 | 0-0.1 | | 0.1-0.1 | | 0.1-0.2 | | 0.1-0.2 | |  |  |
| **BASOPHILS (10^9^/L)** | | **PP** | **FP** | | **FP+7** | | **FP+14** | | **Conv** | |  |  |
| Count | | 5 | 85 | | 66 | | 41 | | 60 | |  |  |
| Mean | | 0.04 | 0.006 | | 0.005 | | 0.002 | | 0.02 | |  |  |
| 95% CI | | -0.03-0.11 | -0.0002-0.01 | | -0.0006-0.01 | | -0.003-0.007 | | 0.008-0.03 | |  |  |
| Half width CI | | 0.07 | 0.01 | | 0.01 | | 0 | | 0.01 | |  |  |
| Median | | 0 | 0 | | 0 | | 0 | | 0 | |  |  |
| Interquartile Range | | 0-0.1 | 0-0 | | 0-0 | | 0-0 | | 0-0 | |  |  |
| **PLATELETS (10^9^/L)** | | **PP** | **FP** | | **FP+7** | | **FP+14** | | **Conv** | |  |  |
| Count | | 8 | 90 | | 70 | | 40 | | 64 | |  |  |
| Mean | | 244 | 206 | | 236 | | 283 | | 237 | |  |  |
| 95% CI | | 203-285 | 195-217 | | 221-251 | | 254-311 | | 222-252 | |  |  |
| Half width CI | | 41.03 | 11.2 | | 14.69 | | 28.28 | | 14.89 | |  |  |
| Median | | 258 | 197 | | 241 | | 270 | | 229 | |  |  |
| Interquartile Range | | 217-279 | 175-231 | | 199-267 | | 226-319 | | 193-283 | |  |  |
| *Continued on next page* | | | | | | | | | | |  |  |
| **RED BLOOD CELLS (10^12^/L)** | | **PP** | **FP** | | **FP+7** | | **FP+14** | | **Conv** | |  |  |
| Count | | 8 | 90 | | 70 | | 41 | | 64 | |  |  |
| Mean | | 5.05 | 4.88 | | 4.8 | | 4.66 | | 4.68 | |  |  |
| 95% CI | | 4.49-5.61 | 4.78-4.99 | | 4.69-4.91 | | 4.53-4.79 | | 4.56-4.79 | |  |  |
| Half width CI | | 0.56 | 0.11 | | 0.11 | | 0.13 | | 0.12 | |  |  |
| Median | | 5.01 | 4.88 | | 4.83 | | 4.72 | | 4.7 | |  |  |
| Interquartile Range | | 4.5-5.64 | 4.49-5.25 | | 4.48-5.08 | | 4.4-4.96 | | 4.33-5.01 | |  |  |
| **HAEMOGLOBIN (g/L)** | | **PP** | **FP** | | **FP+7** | | **FP+14** | | **Conv** | |  |  |
| Count | | 8 | 90 | | 70 | | 41 | | 64 | |  |  |
| Mean | | 152 | 147 | | 145 | | 141 | | 141 | |  |  |
| 95% CI | | 142-162 | 144-150 | | 141-149 | | 137-145 | | 137-144 | |  |  |
| Half width CI | | 10.07 | 3.16 | | 3.87 | | 3.97 | | 3.31 | |  |  |
| Median | | 153 | 150 | | 148 | | 144 | | 141 | |  |  |
| Interquartile Range | | 147-160 | 138-157 | | 137-153 | | 134-151 | | 129-151 | |  |  |
| **MEAN CORPUSCULAR VOLUME (fL)** | | **PP** | **FP** | | **FP+7** | | **FP+14** | | **Conv** | |  |  |
| Count | | 10 | 90 | | 70 | | 41 | | 64 | |  |  |
| Mean | | 90.3 | 90.2 | | 89.9 | | 90.9 | | 90.9 | |  |  |
| 95% CI | | 86.6-93.9 | 89.1-91.3 | | 88.7-91.2 | | 89.8-92.1 | | 89.8-92 | |  |  |
| Half width CI | | 3.66 | 1.1 | | 1.23 | | 1.16 | | 1.12 | |  |  |
| Median | | 90.2 | 91 | | 90.7 | | 91.4 | | 90.9 | |  |  |
| Interquartile Range | | 86-94.5 | 88.2-93.1 | | 87.9-92.8 | | 88-93.2 | | 88.6-93.5 | |  |  |
| **MEAN CORPUSCULAR HAEMOGLOBIN (pg)** | | **PP** | **FP** | | **FP+7** | | **FP+14** | | **Conv** | |  |  |
| Count | | 10 | 90 | | 70 | | 41 | | 64 | |  |  |
| Mean | | 30.5 | 30.2 | | 30 | | 30.4 | | 30.1 | |  |  |
| 95% CI | | 29.1-31.8 | 29.8-30.6 | | 29.6-30.5 | | 29.9-30.8 | | 29.8-30.5 | |  |  |
| Half width CI | | 1.34 | 0.39 | | 0.46 | | 0.43 | | 0.37 | |  |  |
| Median | | 30.5 | 30.4 | | 30.2 | | 30.4 | | 30.1 | |  |  |
| Interquartile Range | | 29-31.9 | 29.6-31.3 | | 29.2-31.3 | | 29.6-31.4 | | 29.3-31.3 | |  |  |
| **MEAN CORPUSCULAR HAEMOGLOBIN CONCENTRATION (g/L)** | | **PP** | **FP** | | **FP+7** | | **FP+14** | | **Conv** | |  |  |
| Count | | 10 | 90 | | 70 | | 41 | | 64 | |  |  |
| Mean | | 337 | 335 | | 330 | | 334 | | 331 | |  |  |
| 95% CI | | 331-343 | 333-337 | | 321-339 | | 331-337 | | 329-333 | |  |  |
| Half width CI | | 5.76 | 1.75 | | 8.84 | | 2.75 | | 1.94 | |  |  |
| Median | | 335 | 335 | | 334 | | 333 | | 332 | |  |  |
| Interquartile Range | | 333-338 | 329-341 | | 327-342 | | 328-340 | | 327-335 | |  |  |
| **MEAN PLATELET VOLUME (fL)** | | **PP** | **FP** | | **FP+7** | | **FP+14** | | **Conv** | |  |  |
| Count | | 8 | | | 86 | | 67 | | 38 | | 61 | |
| Mean | | 8.23 | | | 8.59 | | 8.24 | | 8.31 | | 8.54 | |
| 95% CI | | 7.2-9.25 | | | 8.36-8.82 | | 8.01-8.46 | | 7.9-8.73 | | 8.27-8.82 | |
| Half width CI | | 1.03 | | | 0.23 | | 0.22 | | 0.41 | | 0.27 | |
| Median | | 7.8 | | | 8.6 | | 8.2 | | 8.25 | | 8.3 | |
| Interquartile Range | | 7.53-8.73 | | | 7.88-9.13 | | 7.7-8.8 | | 7.4-8.9 | | 7.9-9.15 | |

**Table S3: Fig2 Mixed-effects model statistical test results.** Statistical analysis was performed using a mixed-effects model (REML) in GraphPad Prism, with multiple comparisons conducted using Dunnett’s post hoc test comparing each timepoint against the final convalescent timepoint. P values were first adjusted for multiple comparisons within each parameter using Dunnett method. To control the family-wise error rate across all 13 parameters, the resulting p-values were further adjusted using the Bonferroni correction (Significant comparisons are indicated as follows: P<0.05 = *, P < 0.01 = **, P < 0.001 = ***, P < 0.0001 = ****. P values with a value <0.0001 were treated as 0.0001 during Bonferroni correction).

| **Group1** | **Group2** | **p.adjusted** | **adj.signif** | **p.bonf** | **p.bonf.signif** | **Cell type** |
| --- | --- | --- | --- | --- | --- | --- |
| Conv | PP | 4.77E-02 | * | 0.6201 |  | White Blood Cells |
| Conv | FP | <0.0001 | **** | 0.0013 | ** | White Blood Cells |
| Conv | FP+7 | 2.3E-03 | ** | 0.0299 | * | White Blood Cells |
| Conv | FP | <0.0001 | **** | 0.0013 | ** | Red Blood Cells |
| Conv | FP+7 | 9E-04 | *** | 0.0117 | * | Red Blood Cells |
| Conv | PP | 2.94E-02 | * | 0.3822 |  | Haemoglobin |
| Conv | FP | <0.0001 | **** | 0.0013 | ** | Haemoglobin |
| Conv | FP+7 | <0.0001 | **** | 0.0013 | ** | Haemoglobin |
| Conv | FP | 1E-04 | *** | 0.0013 | ** | Mean Corpuscular Volume |
| Conv | FP+7 | <0.0001 | **** | 0.0013 | ** | Mean Corpuscular Volume |
| Conv | PP | 1.04E-02 | * | 0.1352 |  | Mean Corpuscular Haemoglobin |
| Conv | FP+14 | 3.8E-02 | * | 0.494 |  | Mean Corpuscular Haemoglobin |
| Conv | PP | 4.18E-02 | * | 0.5434 |  | MCH Concentration |
| Conv | FP | 6E-04 | *** | 0.0078 | ** | MCH Concentration |
| Conv | FP | <0.0001 | **** | 0.0013 | ** | Platelets |
| Conv | FP+14 | <0.0001 | **** | 0.0013 | ** | Platelets |
| Conv | FP | <0.0001 | **** | 0.0013 | ** | Neutrophils |
| Conv | FP+7 | 1.09E-02 | * | 0.1417 |  | Neutrophils |
| Conv | PP | 3.53E-02 | * | 0.4589 |  | Lymphocytes |
| Conv | FP | <0.0001 | **** | 0.0013 | ** | Lymphocytes |
| Conv | FP+7 | 3.5E-03 | ** | 0.0455 | * | Monocytes |
| Conv | FP | <0.0001 | **** | 0.0013 | ** | Eosinophils |
| Conv | FP+7 | <0.0001 | **** | 0.0013 | ** | Eosinophils |
| Conv | FP | 3.15E-02 | * | 0.4095 |  | Basophils |
| Conv | FP+14 | 2.13E-02 | * | 0.2769 |  | Basophils |
| Conv | FP+7 | 1.08E-02 | * | 0.1404 |  | Mean Platelet Volume |

**Table S4: Proportion of study participants that presented with full blood count parameters outside of the normal clinical range.** Fisher’s Exact test was performed to determine the significance between infection timepoints compared to the convalescent timepoint. *Where there are differences in the lower and upper thresholds between males and females, the lowest threshold and highest threshold from either sex was used. PP, pre-positive timepoint; FP, first study-positive; FP+7, seven to ten days after first study-positive; FP+14 fourteen days after first study-positive; Conv, convalescent.

|  | **Normal Threshold** | **Timepoint** | **No. below lower threshold (%)** | ***P-*value** | **No. above upper threshold (%)** | ***P-*value** |
| --- | --- | --- | --- | --- | --- | --- |
| **WBC*** | 4.2x10^9^/L   -  11.2x10^9^/L | PP | 3/10 (30) | 0.11 | 0/10 (0) | 1 |
|  |  | FP | 38/90 (42.2) | **0.0002** | 0/90 (0) | 1 |
|  |  | FP+7 | 12/70 (17.1) | 0.199 | 0/70 (0) | 1 |
|  |  | FP+14 | 3/41 (7.3) | 1 | 0/41 (0) | 1 |
|  |  | Conv | 5/64 (7.8) | - | 0/64 (0) | - |
| **Neutrophils** | 2.0x10^9^/L   -   7.1 x10^9^/L | PP | 1/8 (12.5) | 0.407 | 0/8 (0) | 1 |
|  |  | FP | 28/89 (31.5) | **0.001** | 0/89 (0) | 1 |
|  |  | FP+7 | 11/70 (15.7) | 0.09 | 0/70 (0) | 1 |
|  |  | FP+14 | 1/41 (2.4) | 1 | 0/41 (0) | 1 |
|  |  | Conv | 6/63 (9.5) | - | 0/63 (0) | - |
| **Lymphocytes** | 1.1 x10^9^/L   -   3.6 x10^9^/L | PP | 0/8 (0) | 1 | 0/8 (0) | 1 |
|  |  | FP | 20/89 (22.5) | **<0.0001** | 0/89 (0) | 1 |
|  |  | FP+7 | 2/70 (2.9) | 0.499 | 1/70 (1.4) | 1 |
|  |  | FP+14 | 0/41 (0) | 1 | 0/41 (0) | 1 |
|  |  | Conv | 0/63 (0) | - | 0/63 (0) | - |
| **Monocytes** | 0.3 x10^9^/L   -   0.9 x10^9^/L | PP | 0/8 (0) | 1 | 0/8 (0) | 1 |
|  |  | FP | 2/89 (2.2) | 0.513 | 4/89 (4.5) | 0.649 |
|  |  | FP+7 | 4/70 (5.7) | 0.124 | 0/70 (0) | 0.478 |
|  |  | FP+14 | 2/41 (4.9) | 0.162 | 0/41 (0) | 1 |
|  |  | Conv | 0/63 (0) | - | 1/63 (1.6) | - |
| **Eosinophils** | 0 x10^9^/L   -   0.9 x10^9^/L | PP | - | - | 0/5 (0) | 1 |
|  |  | FP | - | - | 0/84 (0) | 0.421 |
|  |  | FP+7 | - | - | 1/67 (1.49) | 1 |
|  |  | FP+14 | - | - | 0/41 (0) | 1 |
|  |  | Conv | - | - | 1/60 (1.67) |  |
| **Basophils** | 0 x10^9^/L   -   0. x10^9^/L | PP | - | - | 0/5 (0) | 1 |
|  |  | FP | - | - | 0/85 (0) | 1 |
|  |  | FP+7 | - | - | 0/66 (0) | 1 |
|  |  | FP+14 | - | - | 0/41 (0) | 1 |
|  |  | Conv | - | - | 0/60 (0) | - |
| *Continued on next page* | | | | | | |
| **Platelets*** | 130x10^9^/L  -   400x10^9^/L | PP | 0/8 (0) | 1 | 0/8 (0) | 1 |
|  |  | FP | 4/90 (4.4) | 0.649 | 1/90 (1.1) | 1 |
|  |  | FP+7 | 3/70 (4.3) | 0.622 | 2/70 (2.9) | 0.498 |
|  |  | FP+14 | 0/40 (0) | 1 | 4/40 (10) | **0.025** |
|  |  | Conv | 1/64 (1.6) | - | 0/64 (0) | - |
| **RBC*** | 3.73x10^12^/L    -   5.46x10^12^/L | PP | 0/8 (0) | 1 | 2/8 (25) | 0.168 |
|  |  | FP | 1/90 (1.1) | 1 | 13/90 (14.4) | 0.195 |
|  |  | FP+7 | 0/70 (0) | 1 | 7/70 (10) | 0.542 |
|  |  | FP+14 | 1/41 (2.4) | 0.396 | 0/41 (0) | 1 |
|  |  | Conv | 0/64 (0) | - | 4/64 (6.3) | - |
| **Haemoglobin*** | 114g/L    -   168g/L | PP | 0/8 (0) | 1 | 1/8 (12.5) | 0.322 |
|  |  | FP | 2/90 (2.2) | 1 | 4/90 (4.4) | 1 |
|  |  | FP+7 | 2/70 (2.9) | 1 | 4/70 (5.7) | 0.684 |
|  |  | FP+14 | 1/41 (2.4) | 1 | 1/41 (2.4) | 1 |
|  |  | Conv | 1/64 (1.6) | - | 2/64 (3.1) | - |
| **Mean Corpuscular Volume** | 83.5fL  -   99.5 | PP | 1/10 (10) | 0.463 | 0/10 (0) | 1 |
|  |  | FP | 7/90 (7.8) | 0.529 | 1/90 (1.1) | 1 |
|  |  | FP+7 | 4/70 (5.7) | 1 | 1/70 (1.4) | 1 |
|  |  | FP+14 | 2/41 (4.9) | 1 | 0/41 (0) | 1 |
|  |  | Conv | 3/64 (4.7) | - | 1/64 (1.6) | - |
| **Mean Corpuscular Haemoglobin** | 27.5pg -   33.1 | PP | 0/10 (0) | 1 | 1/10 (10) | 0.374 |
|  |  | FP | 4/90 (4.4) | 1 | 2/90 (2.2) | 1 |
|  |  | FP+7 | 5/70 (7.1) | 0.448 | 2/70 (2.9) | 1 |
|  |  | FP+14 | 2/41 (4.9) | 0.646 | 1/41 (2.4) | 1 |
|  |  | Conv | 2/64 (3.1) | - | 1/64 (1.6) | - |
| **Mean Corpuscular Haemoglobin Concentration** | 315g/L  -   350g/L | PP | 0/10 (0) | 1 | 1/10 (10) | 0.27 |
|  |  | FP | 1/90 (1.1) | 1 | 3/90 (3.3) | 0.644 |
|  |  | FP+7 | 1/70 (1.4) | 1 | 2/70 (2.9) | 1 |
|  |  | FP+14 | 0/41 (0) | 1 | 2/41 (4.9) | 0.562 |
|  |  | Conv | 0/64 (0) | - | 1/64 (1.6) | - |
| **Mean  Platelet Volume** | 7.5fL -  11.5fL | PP | 1/8 (12.5) | 1 | 0/8 (0) | 1 |
|  |  | FP | 9/86 (10.5) | 1 | 0/86 (0) | 1 |
|  |  | FP+7 | 14/67 (20.9) | 0.1559 | 0/67 (0) | 1 |
|  |  | FP+14 | 10/38 (26.3) | 0.1002 | 1/38 (2.6) | 0.39 |
|  |  | Conv | 6/61 (9.8) | - | 0/61 (0) | - |

**Supplementary Figures**

**Fig S1: Histograms of full blood count (FBC) variables across all timepoints.** Each panel shows a histogram of the indicated FBC variable, with the y-axis scaled to density. A red line represents the overall density distribution of the data.

**
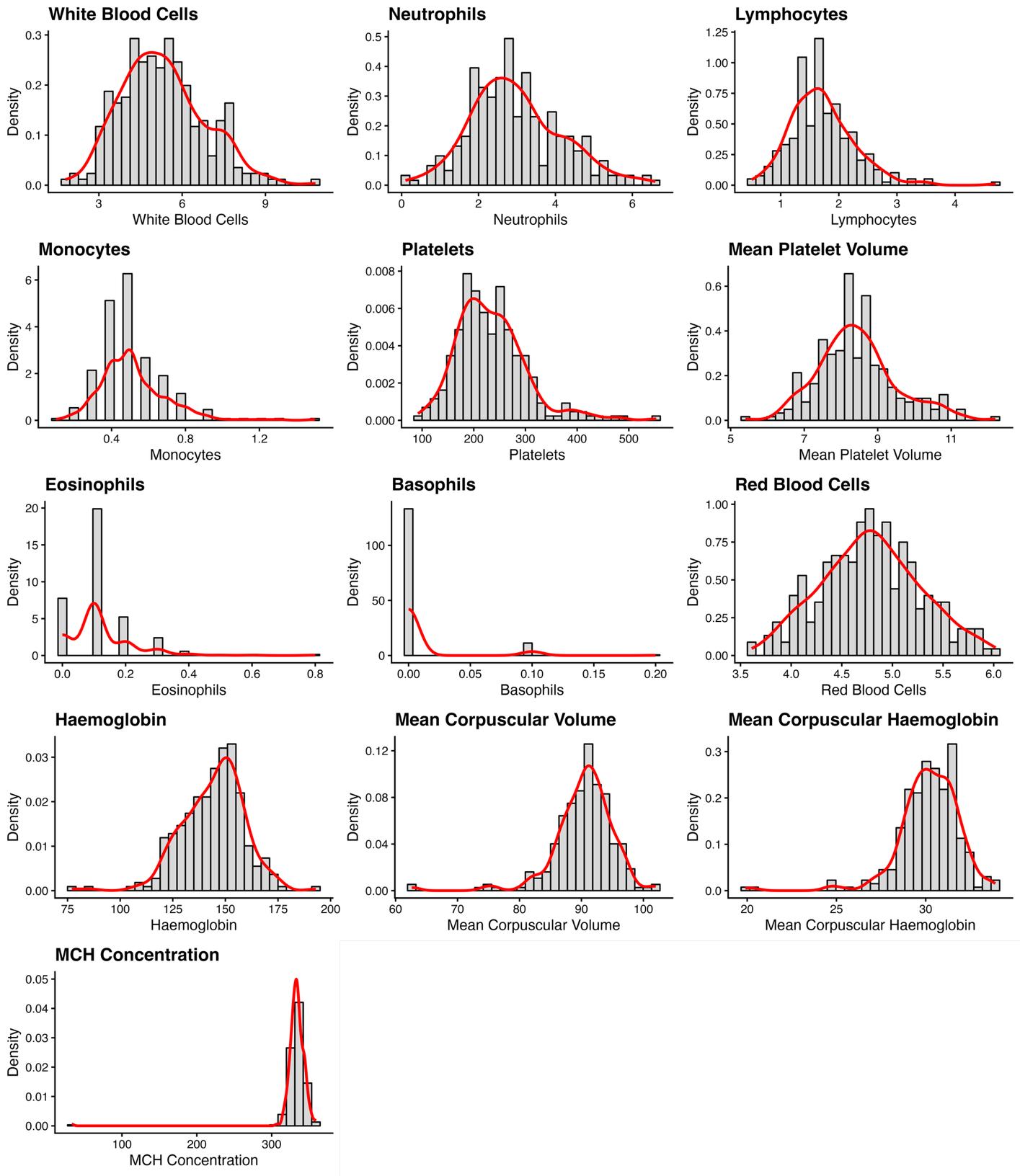
**

**Fig S2: Ridge plots of full blood count (FBC) variables by infection timepoint.** Each panel shows the density distribution of the indicated FBC variable, with separate ridges for each infection timepoint. Mean values are indicated by a quantile line, visualising how the distribution of each variable changes across timepoints.

**
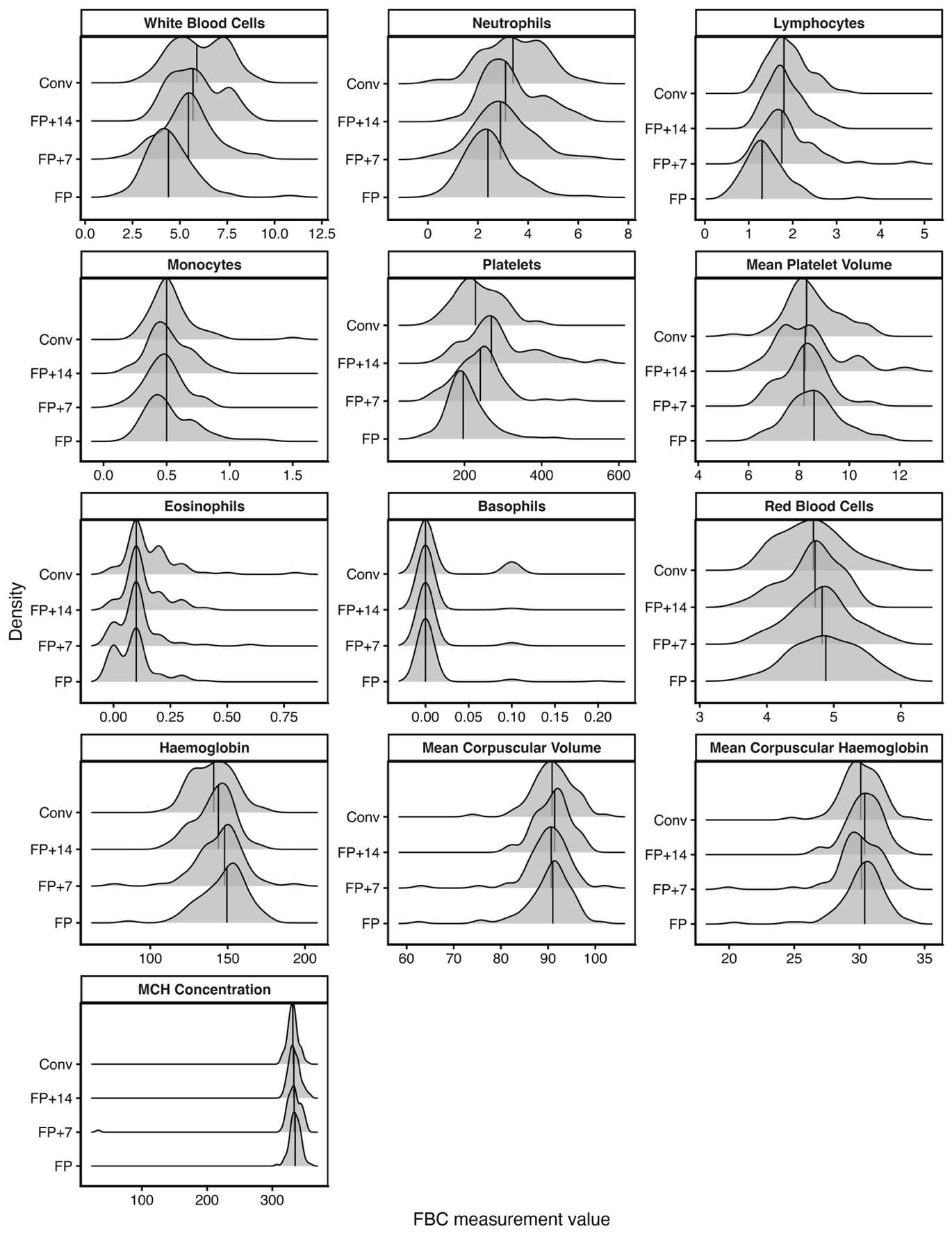
**

**Fig S3:** **The remainder of the longitudinal full blood cell parameter dynamics in 93 cases.** Mean and 95% confidence limits per timepoint of each FBC indicator are depicted by the blue line and shaded area respectively, with individual longitudinal cases behind in grey. The distributions at each timepoint are represented with violin plots. Orange dotted lines indicate the thresholds defined normal ranges from each full blood count parameter. Maximum sample size: PP, pre-positive timepoint (n=10); FP, first study-positive (n=90); FP+7, seven days after first study-positive (n=70); FP+14 fourteen days after first study-positive (n=41); Conv, convalescent (n=64). Mixed effects analysis was adjusted for multiple within parameter comparisons using Dunnett’s multiple comparisons test and subsequently corrected for multiple parameters with Bonferroni correction. Summary statistics including means and 95% confidence limits are listed in Table-S3.


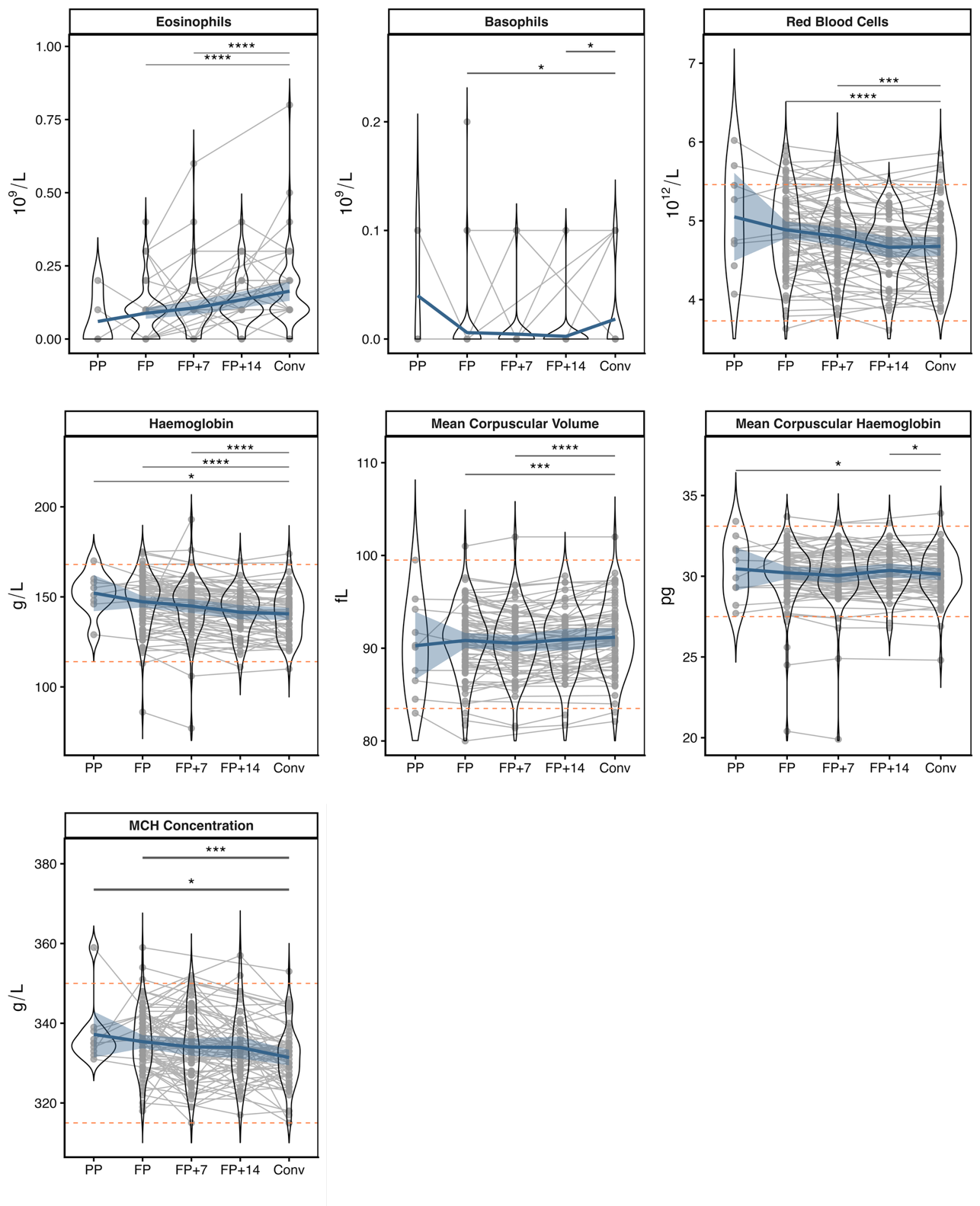


**Fig S4: Correlation between mean platelet volume (MPV) at FP+7 and Platelet count at FP+14.** MPV is significantly negatively correlated with Platelet count (R^2^ = 0.33, R = -0.57, p = 0.0003). Pearsons’s correlation coefficient and p-values were calculated using Pearson’s test. Best-fit lines shown in blue with 95% confidence intervals (grey shaded area) (n = 35).

**
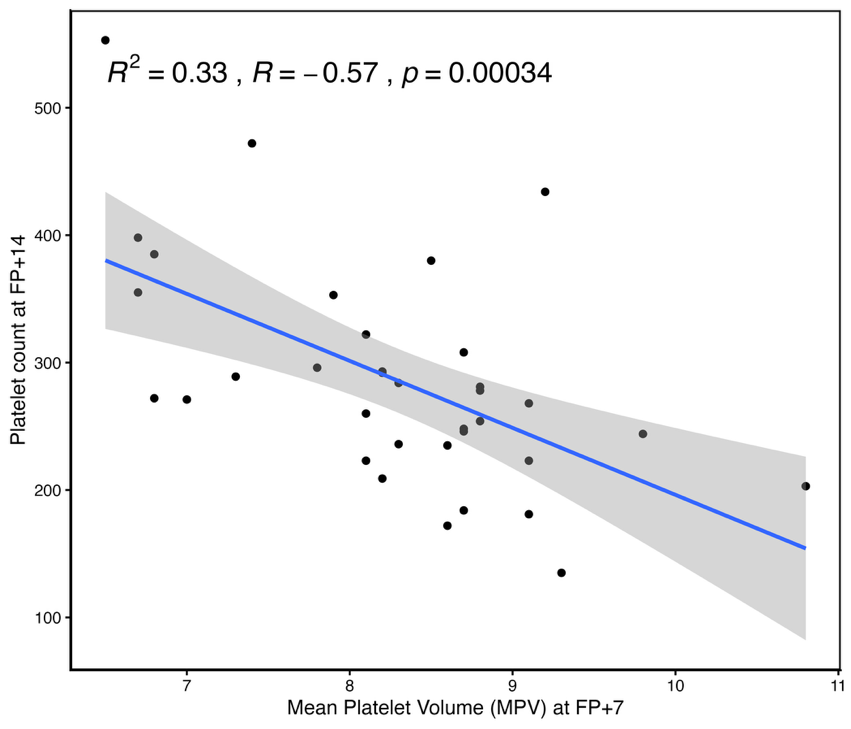
**
