# Supplementary material for: Full blood count dynamics in immunologically naïve individuals with mild COVID-19: A prospective community cohort study
Source: PLoS One. 2026 Jul 9;21(7):e0353142. doi: 10.1371/journal.pone.0353142 (PMC13349189; doi:10.1371/journal.pone.0353142)
Supplement: Attachment — Submitted filename: Response to Reviewers.docx [file pone.0353142.s004.docx]

**Dr Seran Hakki**

**Postdoctoral Research Associate,**

**Depty Lead for Pandemic Preparedness,**

**Academic Career Development Lead.**

NIHR HPRU in Respiratory Infections,

Imperial College London,

National Heart and Lung Institute.

Level 5, VPD Building,

Hammersmith Campus,

London W12 0NN	13 May 2026

**José Ramos-Castañeda M.Sc., Ph.D,
Academic Editor,**

***PLOS ONE***

Dear Dr José Ramos-Castañeda,

***Full blood count dynamics in immunologically naïve individuals with mild COVID-19: a prospective community cohort study***

**Ref: PONE-D-26-10551**

We thank the Academic Editor and the reviewers for their careful evaluation of our manuscript and for the constructive comments provided. We have revised the manuscript accordingly and believe that these changes have substantially improved the clarity and quality of the work.

Below, we provide a detailed point-by-point response to each comment raised.

# Reviewer 1

**1. Reviewer Comment:** Sentence is too long. “Over 30% of cases exhibited transient, clinically defined neutropenia (mean (95% CI): First PCR-Positive (FP) 2.47x109/L (2.26-2.68) vs convalescence 3.34 x109/L (2.98-3.7); p=0.0013), while over 20% exhibited lymphopenia (FP 1.38x109/L (1.28-1.48) vs convalescence 1.79x109/L (1.79-2.01); p=0.0013).’’

**Response:** The abstract results section was revised and split into two shorter statements for improved readability.

**2. Reviewer Comment:** The conclusion of ‘well-characterised’ should be revised as only 30% showed neutropenia & 20% had lymphopenia.

**Response:** Thank you, this has been revised on L46.

**3. Reviewer Comment:** What were the inclusion and exclusion criteria?

**Response:** The inclusion and exclusion criteria for patients providing FBC data are shown in Fig 1A, in the form of a cohort determination flow chart, and are summarised in the Fig 1A legend. They are as follows:

Inclusion criteria:

- Must be ≥12 years old

-Must be unvaccinated pre-enrolment.

-	Must be seronegative for COVID-19 on the data of recruitment

-	Must become PCR positive in the first two recorded timepoints, in order to capture FBC changes during active infection.

-	Must have had symptom onset ≤5 days from the day of recruitment.

-	Must have ≥5 days of longitudinal symptom information.

-	Must have no reported comorbidity.

We have now explicated added this information in the methods section in lines 102-108

**4. Reviewer Comment:** Please define clinically relevant neutropenia.

**Response:** Clinically relevant neutropenia was defined in this study as a neutrophil count < 2x109/L, as outlined in S4 Table.

This reference range, together with the reference ranges used for all other haematological parameters, was set by the clinical laboratory.

We have added in the threshold values used to define clinical parameter ranges within the main text, where clinically abnormal values are discussed.

**5. Reviewer Comment:** It would be good to separate the results & discussion parts for clearer flow of the manuscript.

**Response:** We thank you for this suggestion. We agree that separating the ‘Results’ and ‘Discussion’ sections can improve manuscript flow in some contexts. However, this manuscript is under 2000 words, and combining these sections allowed us to present the findings alongside their interpretation in a concise and integrated manner. An overarching conclusion paragraph has is included to summarise the key findings and implications.

**6. Reviewer Comment:** Line 132-135 – what were the findings of majority of the patients given only 30% had transient neutropenia & 20% lymphopenia?

**Response:** The majority of patients had a lower neutrophil and lymphocyte count at the FP timepoint in comparison to the convalescent timepoint (n = 60). We saw lower neutrophil counts at FP than convalescence in 51/60 participants and lower lymphocyte counts in 55/60 participants.

This has been addressed in the main text, on L172-175.

**7. Reviewer Comment:** Line 144-145 – what was the severity of Covid-19 infection in these patients?

**Response:** The clozapine study comprised a cohort of 31 patients, who had been admitted to the centre for mental illness and had COVID-19 during the study period.

The majority of patients experienced mild COVID-19 (24/31, 77.4%), with only 7 reporting moderate COVID-19 severity (7/31, 22.6%). This additional information has been added to the main text, on L187.

**8. Reviewer Comment:** Lines 152-155 – the p values are similar. Please recheck the results

**Response:** The Bonferroni-adjusted p values were rechecked and confirmed to be correct.

**9. Reviewer Comment:** Remove ‘Unknown 0 (0%)’ from Table S1.

**Response:** This has been amended in the Supplementary Materials.

**10. Reviewer Comment:** What is the relevance of Figure S1? Are all results at all time-points lumped together?

**Response:** In answer to your query, yes, data from all time-points was combined in Figure S1. Visualisation of the overall data distributions was included to provide an overview of the spread, symmetry, and variability of each FBC parameter across the dataset, and to assess whether there were marked departures from approximate normality that would substantially affect the suitability of parametric analyses. While visual inspection alone does not formally establish normality, the combined histograms and ridge plots did not indicate strong skewness or multimodal structure for the principal parameters analysed. Together with the sample size and repeated-measures study design, this supported the use of parametric multiple-comparison testing across timepoints.

Temporal differences between infection-timepoints were subsequently explored using ridge plots in Figure S2, allowing direct visual comparison of distributional shifts across infection and recovery.

**11. Reviewer Comment:** Why was mean & 95% CI used in the figure? At pp interval, the number of cases are consistently less than 30? Would median be more appropriate?

**Response:** Mean and 95% CI were used to facilitate comparison of central tendency and precision across time points, and to align with the statistical framework applied for group-level inference. Although the pp (pre-positive) time point includes fewer than 30 cases, the distributions were not markedly skewed, making the mean an appropriate summary measure, while the confidence intervals provide an interpretable measure of uncertainty.

# Reviewer 2

**1. Reviewer Comment:** It is important to state clearly without any ambiguity in the methodology how the heterogeneity in the sampling timepoints were handled.

It is to be noted that this has to be reported under methodology and not under results and discussion.

**Response:** We thank you for your comment. We have now moved this segment from the results/discussion to the methodology describing how heterogeneity in sampling timepoints relative to infection timing was addressed, now on L101.

While this approach cannot perfectly align all participants to the exact onset of infection due to the sampling frequency, realignment to the first PCR-positive study sample provided the closest achievable approximation. The overall viral trajectories observed across FP-aligned timepoints demonstrated broadly consistent temporal alignment between cases, as shown in Fig 1B.

**2. Reviewer Comment:** It is reported in the abstract that sampling started at the first PCR positivity, but later reported that pre-positive samples were collected. The actual time of sampling initiation have to be reported in the abstract.

**Response:** We apologise for this error. We have corrected the abstract wording on L35.

PCR-positive COVID-19 cases identified by the UK Health Security Agency contact tracing program were approached for recruitment, and sampling was initiated as soon as the first nurse household visit could be performed for the index case and contacts.

Blood samples were collected at D0,7,14,28. Timepoints were subsequently realigned relative to the first PCR-positive study sample (FP). Where the enrolment visit (D0) occurred prior to PCR positivity, these samples were classified as “pre-positive” (PP).

**3. Reviewer Comment:** The discussion of the result focused on FP and convalescence, no picture was given about what happened between those two periods.

The authors have to state whether the 65 individuals were removed from 93 or from 158 (93+65) during Patient recruitment.

**Response:** We thank the reviewer for this comment.

The 65 excluded individuals were removed from the initial cohort of 158 participants prior to derivation of the final analytic dataset; the final study cohort therefore comprises 93 participants. All analyses reported in the manuscript are based on the final cohort of 93 participants, each of whom had data available at the FP timepoint.

Regarding the interpretation of results, we focused the text on statistically supported and clinically relevant changes between defined infection-timepoints, particularly FP and convalescence, while intermediate timepoints (FP7 and FP14) are presented in full within the figures to illustrate the temporal trajectory.

# Reviewer 3

**1. Reviewer Comment:** The main concerns with the current manuscript is the aging of the dataset. This data was collected between 2020 and 2021 and is only submitted for publication in 2026. This creates a question around the current value of the manuscript.

**Response:** The age of the dataset is acknowledged; however, its value lies in the unique temporal context in which it was collected.

These data capture host haematological responses during the early pandemic period, prior to widespread vaccination and the emergence of later variants, providing a relatively unconfounded view of infection dynamics. As such, they serve as a useful reference point for comparison with subsequent cohorts.

While more recent datasets reflect evolving immunity and variant-specific effects, they are inherently more heterogeneous. The present analysis therefore retains relevance by defining baseline responses against which later changes can be interpreted.

**2. Reviewer Comment:** Further to this, the relative small sample size in a proposed longitudinal study of this nature is a limitation.

**Response:** We acknowledge that the sample size is relatively small for a longitudinal study of this nature, which may limit statistical power and the precision of estimates.

The reviewer should also consider the substantial logistical constraints on recruitment and follow-up during a period of national lockdown, including restricted movement, limited access to clinical and research facilities, and the need to minimise in-person contact. These factors directly impacted both enrolment and retention rates.

However, the study design incorporates repeated measures within individuals, allowing each participant to serve as their own control and partially mitigating this limitation. The consistency of observed trends across time points further supports the robustness of the findings, although they should be interpreted with appropriate caution.

**3. Reviewer Comment:** Further to this, in my opinion to classify the study as longitudinal, but only measuring 30 days is questionable. Counts might recover significantly in a longer follow-up time.

**Response:** We agree that longer follow-up could capture additional recovery dynamics. However, the aim of this study was to characterise transient changes in FBC parameters during acute mild infection and early recovery, rather than long-term haematological outcomes.

In this context, a 28-day follow-up captures the clinically relevant window over which acute perturbations are expected to resolve. By this time point, participants are in convalescence and FBC parameters have largely returned towards baseline, allowing us to define both the magnitude and resolution of these short-term changes.

While extended follow-up may provide further insight into complete normalisation in all individuals, this falls outside the primary scope of the current analysis.

Nevertheless, we have removed 6 counts of the word longitudinal throughout the manuscript.

**4. Reviewer Comment:** The major concern though is referring to haematological profiling but only assessing full blood counts. It would have been recommended to assess the coagulation parameters.

**Response:** We agree that inclusion of coagulation parameters would provide a more comprehensive assessment of haematological responses. However, these assays require citrated plasma, and our study protocol collected EDTA blood tube samples, which are not suitable for standard coagulation testing (e.g. PT, aPTT, fibrinogen). As such, coagulation parameters were not measured. Future studies incorporating appropriate sample collection for coagulation assays would be valuable to provide complementary insight into haemostatic changes.

We have revised the terminology used throughout to more precisely reflect the scope of the data (“cellular haematological profiling based on full blood counts”).

We hope that you find the attached revised manuscript suitable for publication in PLOS One.

Thank you once again for your time and expertise in reviewing our work.

Yours sincerely,

**Dr Seran Hakki**

NIHR Health Protection Research Unit in Respiratory Infections

Imperial College London
