# Supplementary material for: Full blood count dynamics in immunologically naïve individuals with mild COVID-19: A prospective community cohort study
Source: PLoS One. 2026 Jul 9;21(7):e0353142. doi: 10.1371/journal.pone.0353142 (PMC13349189; doi:10.1371/journal.pone.0353142)
Supplement: Attachment — Submitted filename: rv 1.docx [file pone.0353142.s002.docx]

This is a prospective community cohort study evaluating the full blood count dynamics in immunologically naïve individuals with mild COVID-19 in the UK.

Please see my recommendations below

Abstract

Sentence is too long. “Over 30% of cases exhibited transient, clinically defined neutropenia (mean (95% CI): First PCR-Positive (FP) 2.47x109/L (2.26-2.68) vs convalescence 3.34 x109/L (2.98-

3.7); p=0.0013), while over 20% exhibited lymphopenia (FP 1.38x109/L (1.28-1.48) vs

convalescence 1.79x109/L (1.79-2.01); p=0.0013).’’
