# Supplementary material for: Full blood count dynamics in immunologically naïve individuals with mild COVID-19: A prospective community cohort study
Source: PLoS One. 2026 Jul 9;21(7):e0353142. doi: 10.1371/journal.pone.0353142 (PMC13349189; doi:10.1371/journal.pone.0353142)
Supplement: Peer review history

# Peer review: Decision Letter 0

**DOI:** 10.1371/journal.pone.0353142.r001

**Authors:** José Ramos-Castañeda

27 Apr 2026

PONE-D-26-10551

Longitudinal full blood count dynamics in immunologically naïve individuals with mild COVID-19: a prospective community cohort study

PLOS One

Dear Dr. Hakki,

Thank you for submitting your manuscript to PLOS ONE. After careful consideration, we feel that it has merit but does not fully meet PLOS ONE’s publication criteria as it currently stands. Therefore, we invite you to submit a revised version of the manuscript that addresses the points raised during the review process.

It is very important that you respond clearly and appropriately to Reviewer 3’s comment.

Please submit your revised manuscript by Jun 11 2026 11:59PM. If you will need more time than this to complete your revisions, please reply to this message or contact the journal office at plosone@plos.org. When you're ready to submit your revision, log on to https://www.editorialmanager.com/pone/ and select the 'Submissions Needing Revision' folder to locate your manuscript file.

Please include the following items when submitting your revised manuscript:

- A letter that responds to each point raised by the academic editor and reviewer(s). You should upload this letter as a separate file labeled 'Response to Reviewers'.
- A marked-up copy of your manuscript that highlights changes made to the original version. You should upload this as a separate file labeled 'Revised Manuscript with Track Changes'.
- An unmarked version of your revised paper without tracked changes. You should upload this as a separate file labeled 'Manuscript'.

If you would like to make changes to your financial disclosure, please include your updated statement in your cover letter. Guidelines for resubmitting your figure files are available below the reviewer comments at the end of this letter.

If applicable, we recommend that you deposit your laboratory protocols in protocols.io to enhance the reproducibility of your results. Protocols.io assigns your protocol its own identifier (DOI) so that it can be cited independently in the future. For instructions see: https://journals.plos.org/plosone/s/submission-guidelines#loc-laboratory-protocols. Additionally, PLOS ONE offers an option for publishing peer-reviewed Lab Protocol articles, which describe protocols hosted on protocols.io. Read more information on sharing protocols at https://plos.org/protocols?utm_medium=editorial-email&utm_source=authorletters&utm_campaign=protocols.

As the corresponding author, your ORCID iD is verified in the submission system and will appear in the published article. PLOS supports the use of ORCID, and we encourage all coauthors to register for an ORCID iD and use it as well. Please encourage your coauthors to verify their ORCID iD within the submission system before final acceptance, as unverified ORCID iDs will not appear in the published article. *Only*  the individual author can complete the verification step; PLOS staff *cannot*  verify ORCID iDs on behalf of authors.

We look forward to receiving your revised manuscript.

Kind regards,

José Ramos-Castañeda, M.Sc., Ph.D

Academic Editor

PLOS One

Journal Requirements:

When submitting your revision, we need you to address these additional requirements.

1. Please ensure that your manuscript meets PLOS ONE's style requirements, including those for file naming. The PLOS ONE style templates can be found at

https://journals.plos.org/plosone/s/file?id=wjVg/PLOSOne_formatting_sample_main_body.pdf and

https://journals.plos.org/plosone/s/file?id=ba62/PLOSOne_formatting_sample_title_authors_affiliations.pdf.

2. Please be informed that funding information should not appear in the Acknowledgments section or other areas of your manuscript. We will only publish funding information present in the Funding Statement section of the online submission form. Please remove any funding-related text from the manuscript.

3. Your ethics statement should only appear in the Methods section of your manuscript. If your ethics statement is written in any section besides the Methods, please delete it from any other section.

4. Please include captions for your Supporting Information files at the end of your manuscript, and update any in-text citations to match accordingly. Please see our Supporting Information guidelines for more information: http://journals.plos.org/plosone/s/supporting-information.

5. If the reviewer comments include a recommendation to cite specific previously published works, please review and evaluate these publications to determine whether they are relevant and should be cited. There is no requirement to cite these works unless the editor has indicated otherwise.

[Note: HTML markup is below. Please do not edit.]

Reviewers' comments:

Reviewer's Responses to Questions

**Comments to the Author**

1. Is the manuscript technically sound, and do the data support the conclusions?

The manuscript must describe a technically sound piece of scientific research with data that supports the conclusions. Experiments must have been conducted rigorously, with appropriate controls, replication, and sample sizes. The conclusions must be drawn appropriately based on the data presented.

Reviewer #1: Partly

Reviewer #2: Yes

Reviewer #3: Partly

**********

2. Has the statistical analysis been performed appropriately and rigorously?

Reviewer #1: Yes

Reviewer #2: Yes

Reviewer #3: Yes

**********

3. Have the authors made all data underlying the findings in their manuscript fully available?

The PLOS Data policy requires authors to make all data underlying the findings described in their manuscript fully available without restriction, with rare exception (please refer to the Data Availability Statement in the manuscript PDF file). The data should be provided as part of the manuscript or its supporting information, or deposited to a public repository. For example, in addition to summary statistics, the data points behind means, medians and variance measures should be available. If there are restrictions on publicly sharing data—e.g. participant privacy or use of data from a third party—those must be specified.

Reviewer #1: Yes

Reviewer #2: Yes

Reviewer #3: Yes

**********

4. Is the manuscript presented in an intelligible fashion and written in standard English?

PLOS ONE does not copyedit accepted manuscripts, so the language in submitted articles must be clear, correct, and unambiguous. Any typographical or grammatical errors should be corrected at revision, so please note any specific errors here.

Reviewer #1: Yes

Reviewer #2: No

Reviewer #3: Yes

**********

5. Review Comments to the Author

Please use the space provided to explain your answers to the questions above. You may also include additional comments for the author, including concerns about dual publication, research ethics, or publication ethics. (Please upload your review as an attachment if it exceeds 20,000 characters)

Reviewer #1: This is a prospective community cohort study evaluating the full blood count dynamics in immunologically naïve individuals with mild COVID-19 in the UK.

Please see my recommendations below

Abstract

1. Sentence is too long. “Over 30% of cases exhibited transient, clinically defined neutropenia (mean (95% CI): First PCR-Positive (FP) 2.47x109/L (2.26-2.68) vs convalescence 3.34 x109/L (2.98-

3.7); p=0.0013), while over 20% exhibited lymphopenia (FP 1.38x109/L (1.28-1.48) vs

convalescence 1.79x109/L (1.79-2.01); p=0.0013).’’

2. The conclusion of ‘well-characterised’ should be revised as only 30% showed neutropenia & 20% had lymphopenia

Materials & method

What were the inclusion & exclusion criteria?

Please define clinically-relevant neutropenia

Results & discussion

It would be good to separate the results & discussion parts for clearer flow of the manuscript

Line 132-135 – what were the findings of majority of the patients given only 30% had transient neutropenia & 20% lymphopenia?

Line 144-145 – what was the severity of Covid-19 infection in these patients?

Lines 152-155 – the p values are similar. Please recheck the results

Table S1

Ethnicity . to remove unknown 0 (0%)

What is the relevance of Fig S1? Are all results at all time-points lumped together?

Fig 2. Why was mean & 95% CI used in the figure? At pp interval, the number of cases are consistently less than 30? Would median be more appropriate?

Reviewer #2: The authors have made a good attempt at trying to prospectively study the Longitudinal full blood count dynamics in immunologically naïve individuals with mild COVID-19, but i have the following comments:

It is important to state clearly without any ambiguity in the methodology how the heterogeneity in the sampling timepoints were handled.It is to be noted that this has to be reported under methodology and not under results and discussion.

It is reported in the abstract that sampling started at the first PCR positivity,but later reported that prepositive samples were collected.The actual time of sampling initiation have to be reported in the abstract.

The discussion of the result focussed on FP and convalescence, no picture was given about what happened between those two periods.

The authors have to state whether the 65 individuals were removed from 93 or from 158 (93+65) during Patient recruitment.

Reviewer #3: The main concerns with the current mansucript is the aging of the dataset. This data was collected between 2020 and 2021 and is only submitted for publication in 2026. This creates a question around the current value of the mansuscript. Further to this, the relative small sample size in a proposed longitudinal study of this nature is a limitation. Further to this, in my opinion to classify the study as longitudinal, but only measuring 30 days is questionable. Counts might recover significantly in a longer follow-up time. The major concern though is referring to haematological profiling but only assessing full blood counts. It would have been recommended to assess the coagulation parameters.

**********

6. PLOS authors have the option to publish the peer review history of their article (what does this mean?). If published, this will include your full peer review and any attached files.

If you choose “no”, your identity will remain anonymous but your review may still be made public.

**Do you want your identity to be public for this peer review?**  For information about this choice, including consent withdrawal, please see our Privacy Policy.

Reviewer #1: No

Reviewer #2: No

Reviewer #3: No

**********

[NOTE: If reviewer comments were submitted as an attachment file, they will be attached to this email and accessible via the submission site. Please log into your account, locate the manuscript record, and check for the action link "View Attachments". If this link does not appear, there are no attachment files.]

To ensure your figures meet our technical requirements, please review our figure guidelines: https://journals.plos.org/plosone/s/figures

You may also use PLOS’s free figure tool, NAAS, to help you prepare publication quality figures: https://journals.plos.org/plosone/s/figures#loc-tools-for-figure-preparation.

NAAS will assess whether your figures meet our technical requirements by comparing each figure against our figure specifications.

Attachment Submitted filename: rv 1.docx

# Author comment: Author response to Decision Letter 1

**DOI:** 10.1371/journal.pone.0353142.r002

14 May 2026

Please refer to "Response to Reviewers" letter which includes a point by point response

Attachment Submitted filename: Response to Reviewers.docx

# Peer review: Decision Letter 1

**DOI:** 10.1371/journal.pone.0353142.r003

**Authors:** José Ramos-Castañeda, José Ramos-Castañeda

19 Jun 2026

Full blood count dynamics in immunologically naïve individuals with mild COVID-19: a prospective community cohort study

PONE-D-26-10551R1

Dear Dr. Hakki,

We’re pleased to inform you that your manuscript has been judged scientifically suitable for publication and will be formally accepted for publication once it meets all outstanding technical requirements.

Within one week, you’ll receive an e-mail detailing the required amendments. When these have been addressed, you’ll receive a formal acceptance letter and your manuscript will be scheduled for publication.

An invoice will be generated when your article is formally accepted. Please note, if your institution has a publishing partnership with PLOS and your article meets the relevant criteria, all or part of your publication costs will be covered. Please make sure your user information is up-to-date by logging into Editorial Manager at Editorial Manager® and clicking the ‘Update My Information' link at the top of the page. For questions related to billing, please contact billing support.

If your institution or institutions have a press office, please notify them about your upcoming paper to help maximize its impact. If they’ll be preparing press materials, please inform our press team as soon as possible -- no later than 48 hours after receiving the formal acceptance. Your manuscript will remain under strict press embargo until 2 pm Eastern Time on the date of publication. For more information, please contact onepress@plos.org.

Kind regards,

José Ramos-Castañeda, M.Sc., Ph.D

Academic Editor

PLOS One

Additional Editor Comments:

After giving the matter careful consideration, and bearing in mind that Reviewer 4 did not in fact raise any objections to the manuscript itself, but rather offered a general opinion on the manuscript, and, finally, given that the arguments put forward by the three reviewers of the first version have been satisfactorily addressed, I have decided not to forward Reviewer 4’s comments and to accept the manuscript for publication.

Reviewers' comments:

Reviewer's Responses to Questions

**Comments to the Author**

1. If the authors have adequately addressed your comments raised in a previous round of review and you feel that this manuscript is now acceptable for publication, you may indicate that here to bypass the “Comments to the Author” section, enter your conflict of interest statement in the “Confidential to Editor” section, and submit your "Accept" recommendation.

Reviewer #2: All comments have been addressed

Reviewer #4: All comments have been addressed

**********

2. Is the manuscript technically sound, and do the data support the conclusions?

The manuscript must describe a technically sound piece of scientific research with data that supports the conclusions. Experiments must have been conducted rigorously, with appropriate controls, replication, and sample sizes. The conclusions must be drawn appropriately based on the data presented.

Reviewer #2: Yes

Reviewer #4: No

**********

3. Has the statistical analysis been performed appropriately and rigorously?

Reviewer #2: Yes

Reviewer #4: No

**********

4. Have the authors made all data underlying the findings in their manuscript fully available?

The PLOS Data policy requires authors to make all data underlying the findings described in their manuscript fully available without restriction, with rare exception (please refer to the Data Availability Statement in the manuscript PDF file). The data should be provided as part of the manuscript or its supporting information, or deposited to a public repository. For example, in addition to summary statistics, the data points behind means, medians and variance measures should be available. If there are restrictions on publicly sharing data—e.g. participant privacy or use of data from a third party—those must be specified.

Reviewer #2: Yes

Reviewer #4: Yes

**********

5. Is the manuscript presented in an intelligible fashion and written in standard English?

PLOS ONE does not copyedit accepted manuscripts, so the language in submitted articles must be clear, correct, and unambiguous. Any typographical or grammatical errors should be corrected at revision, so please note any specific errors here.

Reviewer #2: Yes

Reviewer #4: Yes

**********

6. Review Comments to the Author

Please use the space provided to explain your answers to the questions above. You may also include additional comments for the author, including concerns about dual publication, research ethics, or publication ethics. (Please upload your review as an attachment if it exceeds 20,000 characters)

Reviewer #2: The authors have adequately addressed the comments made at the initial review. The comment dwelt mainly on the sampling method, which the authors have made more explicit, hence to account for heterogeneity in the day of enrolment relative to the day of acquiring detectable infection, sampling timepoints were realigned relative to the day of the first PCR-positive study sample “Pre-positive” (PP) refers to blood samples taken prior to the case testing PCR-positive, whilst “First PCR-positive” (F refers to the blood samples taken when the case first tested PCR-positive in

the study. “FP+7” and “FP+14” refers to samples taken seven- and fourteen-days post-FP respectively.

Reviewer #4: 1. Introduction: Authors could be introduce major information about pathophysiology and biological explication why could COVID affect blood counts?

2. Methodology: That is curious you evaluated patients and cell blood counts after to take of exam, but for me could be more relevant include count cell since starting symptoms, because of an information bias. Additionally you don't mention size necessary for this paper.

3. Results and conclusion: It is interesting your results but I don't understand what is clinical relevance to take of decisions with this results, how this results could be help to apply in daily practice.

**********

7. PLOS authors have the option to publish the peer review history of their article (what does this mean?). If published, this will include your full peer review and any attached files.

If you choose “no”, your identity will remain anonymous but your review may still be made public.

**Do you want your identity to be public for this peer review?**  For information about this choice, including consent withdrawal, please see our Privacy Policy.

Reviewer #2: No

Reviewer #4: No

**********

# Editor report: Acceptance letter

**DOI:** 10.1371/journal.pone.0353142.r004

**Authors:** José Ramos-Castañeda, José Ramos-Castañeda

PONE-D-26-10551R1

PLOS One

Dear Dr. Hakki,

I'm pleased to inform you that your manuscript has been deemed suitable for publication in PLOS One. Congratulations! Your manuscript is now being handed over to our production team.

At this stage, our production department will prepare your paper for publication. This includes ensuring the following:

* All references, tables, and figures are properly cited

* All relevant supporting information is included in the manuscript submission,

* There are no issues that prevent the paper from being properly typeset

You will receive further instructions from the production team, including instructions on how to review your proof when it is ready. Please keep in mind that we are working through a large volume of accepted articles, so please give us a few days to review your paper and let you know the next and final steps.

Lastly, if your institution or institutions have a press office, please let them know about your upcoming paper now to help maximize its impact. If they'll be preparing press materials, please inform our press team within the next 48 hours. Your manuscript will remain under strict press embargo until 2 pm Eastern Time on the date of publication. For more information, please contact onepress@plos.org.

You will receive an invoice from PLOS for your publication fee after your manuscript has reached the completed accept phase. If you receive an email requesting payment before acceptance or for any other service, this may be a phishing scheme. Learn how to identify phishing emails and protect your accounts at https://explore.plos.org/phishing.

If we can help with anything else, please email us at customercare@plos.org.

Thank you for submitting your work to PLOS ONE and supporting open access.

Kind regards,

PLOS ONE Editorial Office Staff

on behalf of

Dr. José Ramos-Castañeda

Academic Editor

PLOS One
